# Report of Committee on Practical Medicine

**Published:** 1864

**Authors:** N. S. Davis

**Affiliations:** Prof. of Practical and Clinical Medicine


### CHICAGO MEDICAL EXAMINER.

N. S. DAVIS, M.D., Editor.

VOL. V. AUGUST, 1864. NO. 8.

### Original Contributions.

ARTICLE XXXI.

### REPORT OF COMMITTEE ON PRACTICAL MEDICINE.

By N. S. DAVIS, M.D., Prof. of Practical and Clinical Medicine.

Presented to the Illinois State Medical Society, May, 1864.

(*Concluded.*)

*Improvements.*—Having devoted so large a space to the character of such epidemics as have prevailed in this State during the past year, the consideration of this branch of our report may be brief.

There are three principal modes by which Practical Medicine may be improved, viz:—

First, by a clearer knowledge of the indications to be fulfilled by treatment, afforded by more exact knowledge of etiology, pathology, and diagnosis.

Second, by new applications of remedies already more or less known to the profession.

Third, by the discovery and application of new remedial agents, and the determination of their value in the treatment of diseases.

It needs no comments to show that every addition to our knowledge of the causes capable of inducing morbid action, aids us in understanding the nature of the action thereby induced. Equally evident is the fact, that the more accurately we can determine, not only, the location, extent, and nature of diseases, but also the stage of their advancement, the more exactly and successfully shall we be able to adjust the use of remedies in their treatment. It is by improvements in these respects, coupled with increased knowledge of the *modus operandi* of medicines, that we are enabled to establish the practice of medicine on a rational, instead of empirical basis. When the physician has so closely studied the symptoms, physiognomy, nature, and tendencies of diseases, that he is able to comprehend clearly the exact pathological condition of his patient, by a very simple process of reasoning, he determines what changes are necessary to arrest the morbid and re-establish the healthy actions of the affected tissues or organs. In other words, he sees clearly the indications it is desirable to fulfill, or the exact objects to be accomplished by treatment. With a corresponding knowledge of the properties and effects of remedial agents, he is even capable of promptly selecting such agent or combination of agents as will meet the indications in the individual case before him. He thus becomes not only a rational, but also, an independent practitioner. He is rational, because he prescribes medicinal agents to accomplish definite and clearly perceived purposes in retarding or arresting morbid action; and independent, because instead of relying on the particular formulæ of his books or teachers, he is at all times able to choose, from the materia medica, such agents and combinations as are adapted to each case as it is presented before him. Hence, if those who seek to improve Practical Medicine would cultivate more patiently and rigidly the departments of etiology, pathology, and diagnosis, more substantial advancement would be made, than in the search after new remedies. In regard to the first of these departments, we need much more extended and minute observations in regard to the hygrometrical, thermometrical, and electrical conditions of the atmosphere in connection with the prevalence of epidemic and endemic diseases. We need more extensive and careful observations in regard to the connection between the mental and physical habits of individuals and classes of individuals, and the diseases with which they are most afflicted. To aid in all these investigations, we greatly need, also, a reliable registry of deaths throughout the whole State, based on the certificates of responsible physicians. In the department of pathology proper, the investigations have been active, and have made substantial progress in some directions.

The histology of morbid products, and the morbid conditions of the blood and secretions, have specially attracted the attention of the profession both in Europe and America. In June, 1863, Prof. E. ANDREWS, a member of this Society, presented to the meeting of the American Medical Association, in this city, a paper on "Diathesis, and their surgical relations," in which pathological views of much importance were set forth. The object of the writer was, to show that erysipelas, both idiopathic and traumatic, is essentially dependent on excessive alkalinity of the blood and fluids; and that a judicious use of mineral acid tonics, especially the tincture of chloride of iron, is capable, not only, of curing the disease, but also, of acting the part of an efficient prophylactic.

During the past year, Dr. REID, of Chicago, read to the Chicago Medical Society an interesting paper on catalytic changes in the blood, more especially in the class of diseases usually called zymotic. And the subject of blood-poisoning, as a source of disease has been frequently under discussion in the same Society. The great attention which has been paid to the histology of morbid products, and to the morbid conditions of the fluids, has been the natural result of advances in organic chemistry, and the general use of the microscope in aid of pathological, as well as physiological, researches. Indeed, so far have these influences swayed the professional mind of the present time, that a modified *humoral pathology* has again become predominant; and the words "Blood Poisons," and "Blood Diseases," have become almost as prominent in the medical literature of the present, as the words *concoction*, *fermentation*, *humors*, *&c.*, were at an early period in the history of our art. While we do not object to any amount of investigations in the directions here alluded to, nor deny but that important diseases may arise from subtle poisons introduced into the blood, we do protest against the ready assumption (without any adequate proof) that such poisons actually exist and constitute the efficient causes of almost all of the acute diseases with which our patients are afflicted.

It must not be forgotten, that many of the morbid conditions and products found in the blood are the *consequences* rather than the *causes* of disease; and that the organized structures are endowed with certain properties, capable of being modified so as to constitute primary morbid conditions. As these properties are intimately connected with the processes of nutrition, disintegration, calorification, and secretion, any morbid alterations of them would necessarily alter, also, the functions with which they are connected; and the latter would equally induce changes in the blood and secretions. We regret to see so prevalent a disposition among medical writers to *assume* the existence of causes of disease, whether such causes are called *blood-poisons* or not, and then boldly apply them in explanation of morbid actions and in their deductions for treatment, as though the existence and properties of the poisons or causes had been fully demonstrated. We regret it, because it is contrary to all sound rules of philosophical research, and because it is so well calculated to mislead or deceive the student. No system of pathology will be found correct, or capable of permanent sway over the minds of the profession, that does not recognize primary morbid changes or elementary forms of disease both in the fluids and solids of the human body, and make a careful discrimination between those morbid conditions of the blood which are primary and dependent on the introduction of poisonous agents from without, and those that are secondary or the result of primary changes in the properties and actions of the solids. In studying the subjects of etiology, pathology, and diagnosis, the field is so extensive, the elements or conditions involved so various, and the nature of all processes in living structures so intricate, that it requires a constant effort of the mind to avoid the adoption of conclusions founded on insufficient data, or drawn from only a partial investigation. Nothing would contribute more to the advancement of practical medicine, than a more complete and patient observation of facts, and the avoidance of too hasty generalizations.

In regard to the new application of old remedies, we have but few items worthy of mention. One of the most troublesome and distressing conditions attendant upon pregnancy, especially in the earlier part of the period, is a great irritability of the gastric nerves, inducing an almost constant sense of nausea and frequent vomiting. This is sometimes continued to such a degree as to deprive the patient of food, until to the suffering is added a dangerous degree of anemia, or impoverishment of the blood.

Believing the gastric symptoms in these cases to be simply a reflex or sympathetic irritation, derived directly from a morbidly sensitive condition of the uterine nerves, I have been induced to use as remedies, both anesthetics and a certain class of narcotics. The former only in extreme cases, but the latter frequently. The narcotic which I have found most beneficial is atropine; and the best form of administration, the sugar coated pills of one fiftieth of a grain taken about twenty minutes before each meal. This form is the best because the dose is sufficient, and it can be taken without the slightest feeling of disgust or inconvenience to the patient.

The first case in which I resorted to the use of chloroform, was one of great severity. The woman was in the seventh month of pregnancy. She had been almost constantly under the depressing influence of nausea from the commencement of her pregnancy, and had rejected by vomiting so large a proportion of all the food taken, that she had become extremely anemic. For several weeks previous to my visit, she had been wholly unable to leave her bed, and so exhausted from nausea and vomiting, that the propriety of inducing premature labor had been seriously considered by her medical attendants. By giving her the sugar coated granules of atropine, at first every four hours, and subsequently at every meal-time, causing her to inhale four or five full inspirations of the vapor of chloroform immediately before taking nourishment, the vomiting was prevented, and she gradually recovered, and passed through her confinement at the proper time. The same means have been found effectual in several other cases. The chloroform is given only to the extent of producing a moderately soothing effect on the sensibility of the par vagum, and may be given not only immediately before swallowing nourishment, but repeated at intervals of five or ten minutes for half an hour afterwards. In no case have I carried it to the extent of producing anæsthesia or insensibility.

*Nausea from Opiates.*—It is well known that one of the most serious inconveniences resulting from the use of opiates in many patients, consists in a protracted and depressing nausea with frequent vomiting, after the first narcotic effects have began to subside. During the last two years, I have seen several cases of this kind in which almost entire relief was obtained by two doses of atropine or belladonna, given at an interval of one hour. And there are probably very few patients who could not receive the benefit of opiates in attacks where they are strongly indicated, if the attending physician should provide them with suitable doses of belladonna to take at the proper interval after the exhibition of the opiate. Another very unpleasant effect of opiates, in some patients, is an excited sleepless delirium with contracted pupils. This may be speedily and almost certainly relieved by moderate doses of belladonna. The use of this remedy for correcting the unpleasant cerebral symptoms, induced by opiates used in the treatment of the bowel affections of children, is of great value.

*Chlorate of Potassa in Paralysis.*—I have occasionally met with cases of paralysis, that appeared to be caused by deficient oxygenation and decarbonization of the blood; thereby inducing direct loss of sensibility in portions of the nervous system. The first case of this kind that attracted my attention was a child two years old, with congenital cyanosis, from what I regarded as an open ductus arteriosus. The child's lips and nails were constantly purple, and after sleeping in a close room, it was found to have complete *hemaphlegia.* There was no fever or the slightest indications of determination of blood to the brain.

Regarding the paralysis as the result of imperfect decarbonization of the blood and the deficient stimulus of oxygen, I gave it chlorate of potassa dissolved in mucilage of gum arabic as freely as it would bear without danger of inducing irritation of the mucous membranes. After the first two days, very small doses of strychnine and citrate of iron were given in addition to the chlorate of potassa. Within twenty-four hours after commencing the treatment, the color of the lips and ends of the fingers began to improve; and in two weeks the paralysis had disappeared. The child lived about one year afterwards, and died under symptoms of cholera morbus. Another case of recent occurrence is that of an adult, male, aged about 25 years. He had been affected with cough and increasing emaciation for two years. He was brought into the Mercy Hospital with complete hemaphlegia of the right side. He was considerably emaciated; his lips purple, or leaden color; his pulse soft and weak; his skin cool and relaxed; his respirations short and frequent, with a somewhat harassing cough, accompanied by free muco-purulent expectoration. His appetite was bad, but bowels nearly regular. A physical examination showed extensive dullness on percussion over the right infraclavicular and mammary regions, and slight dullness directly below the clavicle on the left side. There was also increased vibration of voice in the same regions; and prolonged, rough, and irregular respiratory murmur with a coarse crepitant or sub-mucous rhonchus in the upper lobe of the right lung. These physical signs, in connection with the general symptoms and history of the case, left no doubt about the existence of extensive tubercular deposits in the right lung, with some points of softening, and also a smaller amount of deposit in the apex of the left lung. The capacity of the lungs for air being thus seriously diminished, the blood was deficiently arterialized, giving rise to the leaden hue of the lips, the shortness of breath, general muscular weakness, and finally to the paralysis. In this case I directed a liberal use of the chlorate of potassa dissolved in water, and a mixture of glycerine--℥iii, syrup iodide of iron--℥i, and sulphate of morphia--2grs., of which a teaspoonful was given four times a day. Although paralysis of motion in the right arm and leg was complete at the time of his admission to the hospital, yet under the above treatment it improved so rapidly that in two weeks he could walk with the leg and feed himself with the hand. Several cases of paralysis, of the same pathological character, have come under my care in which there was no pulmonary or other organic disease. They were in laboring men, who were in the habit of using tobacco and alcoholic drinks somewhat freely, and sleeping in small, dirty, and unventilated rooms. The paralysis in all these cases came on in the night, without being preceded or accompanied by any febrile or inflammatory symptoms. Neither was there any subsequent reaction; but on the contrary, the pulse remained soft and slow; skin cool and muscles flaccid; bowels regular, and appetite moderate. All these cases recovered under the influence of good air; nutritious but unstimulating diet; aided by the internal use of chlorine salts and small doses of strychnine.

*New Remedies.*—The chief articles introduced into practice in this State, during the past two years, as new remedies, are, the sulphites of soda and lime; bromine and bromide of ammonium; iodide of lime; and permanganate of potassa.

The prominence which supposed *blood-poisons* have assumed, in the more recent pathological discussions, has led to a search for such remedial agents as were supposed to be efficient either in neutralizing the poisons or in preventing their septic influence on the constituents of the blood. With this purpose, Dr. A. FISHER, of this city, first used internally the sulphites of soda and lime, in cases of gangrene and erysipelas. The results of his cases, and many others occurring in my own practice, are familiar to nearly all the members of the Society, through the pages of the *Chicago Medical Examiner.* I have used the sulphites in a considerable number of cases of malignant erysipelas; the more severe and malignant forms of small-pox, and scarlet fever; in puerpural fever; and in a few cases of cerebro-spinal meningitis. From the trials thus far made, I am induced to believe that when these salts are present in the blood in considerable quantity, they exert a strong influence in counteracting the effects of such poisons as are liable to be absorbed from gangrenous and suppurating surfaces. Hence in all those cases of injury or mechanical violence, followed by sufficient destruction of tissue or suppuration to endanger the absorption of pus or decomposing animal matter; in the suppurative stage of severe variola, there is danger from re-absorption of pus and virus; in the retention of clots or decaying animal matter in the uterus endangering uterine phlebitis, &c., the sulphites will be found valuable remedies. But to be effectual they must be administered early and liberally. How far their administration, during the incubative stage of eruptive fevers, would modify the severity or progress of those affections, I have not had sufficient opportunities to determine. Bromine, as a remedy used locally in hospital gangrene, has been highly recommended by many connected with the military hospitals. Having had little or no personal experience in its use, I must refer the Society to the various medical periodicals for information on the subject.

The attention of the Chicago Medical Society was first called to the use of bromide of ammonium, in certain uterine and nervous affections, by Dr. I. HATCH, of this city. It has been found most useful in chorea, epilepsy, and those severe headaches connected with irritation in the neck of the uterus. It may be given to adults in doses of from three to ten grains. The iodide of lime has been proposed and used as a substitute for the iodide of potassa. It is claimed that it possesses all the efficacy of the iodide of potassa in the treatment of disease, while it is much cheaper, less apt to irritate the stomach and bowels, and more pleasant to take.

Permanganate of potassa, has been recommended to the profession as an internal remedy for the same class of diseases as the bromide of ammonium; while locally it is used for ill-conditioned ulcers, &c. It has also been recently highly recommended by a physician of Ohio, as a remedy in the treatment of cerebro-spinal meningitis or spotted fever. He gave it in doses of from a quarter to half a grain every one, two, or three hours. It is a remedy well worth further trials in the whole class of cerebro-spinal and nervous affections, as well as in those diseases supposed to depend on blood-poisoning.

I had intended to discuss more at length the *modus operandi* of some of the foregoing medicines, but the unexpected length of this report precludes further comments.

